# Prevention and Treatment of Osteoporosis Using Chinese Medicinal Plants: Special Emphasis on Mechanisms of Immune Modulation

**DOI:** 10.1155/2018/6345857

**Published:** 2018-02-20

**Authors:** Hongyan Zhao, Ning Zhao, Peng Zheng, Xiaohong Xu, Meijie Liu, Dan Luo, Huihui Xu, Dahong Ju

**Affiliations:** ^1^Experimental Research Center, China Academy of Chinese Medical Sciences, Beijing 100700, China; ^2^Institute of Basic Theory for Chinese Medicine, China Academy of Chinese Medical Science, Beijing 100700, China; ^3^Institute of Clinical Basic Medicine, China Academy of Chinese Medical Sciences, Beijing 100700, China; ^4^Jilin Provincial Hospital of Traditional Chinese Medicine, Changchun University of Chinese Medicine, Changchun 130021, China; ^5^Changchun University of Chinese Medicine, Changchun 130117, China; ^6^Traditional Chinese Medicine Hospital of Changping District, Beijing 102200, China

## Abstract

Numerous studies have examined the pathogenesis of osteoporosis. The causes of osteoporosis include endocrine factors, nutritional status, genetic factors, physical factors, and immune factors. Recent osteoimmunology studies demonstrated that the immune system and immune factors play important regulatory roles in the occurrence of osteoporosis, and people should pay more attention to the relationship between immunity and osteoporosis. Immune and bone cells are located in the bone marrow and share numerous regulatory molecules, signaling molecules, and transcription factors. Abnormal activation of the immune system alters the balance between osteoblasts and osteoclasts, which results in an imbalance of bone remodeling and osteoporosis. The incidence of osteoporosis is also increasing with the aging of China's population, and traditional Chinese medicine has played a vital role in the prevention and treatment of osteoporosis for centuries. Chinese medicinal plants possess unique advantages in the regulation of the immune system and the relationships between osteoporosis and the immune system. In this review, we provide a general overview of Chinese medicinal plants in the prevention and treatment of osteoporosis, focusing on immunological aspects.

## 1. Introduction

Osteoporosis is a systemic skeletal disease characterized by a decrease in bone mass and microstructural degradation, which may increase bone fragility and fracture danger and consequently cause serious complications [1]. The etiology of osteoporosis is primarily concentrated in endocrine and metabolic disorders. However, osteoimmunology demonstrated that the immune system and immune factors may be involved in the development of osteoporosis and play important regulatory roles [2, 3]. The microenvironment created by bone cells provides conditions for development of the immune system, which regulates bone metabolism via B cells, T cells, dendritic cells, and many cytokines [2–5]. Abnormal activation of the immune system alters the balance between osteoblasts and osteoclasts, which results in an imbalance in bone remodeling and causes osteoporosis [6–8].

Osteoporosis seriously affects human health, and there is no safe and effective method to restore lost bone. However, Chinese medicinal plants reduce bone loss and treat osteoporosis [9, 10]. Chinese medicinal plants possess unique advantages in the regulation of the immune system [11–18] and alter the relationships between osteoporosis and the immune system [3, 4, 7]. This review summarizes the progress of Chinese medicinal plants in the prevention and treatment of osteoporosis, focusing on the immunological aspects.

## 2. Immune System and Osteoporosis

The immune system is an important regulator of bone turnover. Therefore, the relationship between the immune system and bone is generally defined as “osteoimmunology.” Osteoimmunology is a new interdisciplinary research field that focuses on the interactions between the immune system and bone at the anatomical, vascular, cellular, and molecular levels [19–21]. Osteoimmunology provides new pathogenetic and clinical interpretations for osteoporosis [6]. Many signaling factors and cytokines are involved in this field [22]. However, osteoprotegerin (OPG)/the receptor activator of nuclear factor-kappa B (RANK)/RANK ligand (RANKL) signaling is a key vinculum that is essential for osteoclastogenesis and immune regulation [4, 23]. Proinflammatory cytokines, such as tumor necrosis factor- (TNF-) *α*, interleukin- (IL-) 1, and IL-17, also play pivotal roles in osteoimmunology [24]. Therefore, abnormalities of the immune system may cause bone diseases, such as osteoporosis and inflammatory bone loss [25]. Balancing the immune response promotes the formation of bone tissue, so as to improve osteoporosis.

### 2.1. RANK/RANKL/OPG Signaling Pathway

A functional imbalance between osteoblasts and osteoclasts causes osteoporosis. As a hallmark of osteoporosis, the increased activation of osteoclasts results in the progressive loss of bone mass and an increased susceptibility to bone fractures. RANKL is a TNF family cytokine that is primarily expressed by osteoblasts and also secreted by bone marrow stromal cells (BMSCs), preosteoblasts, osteocytes, fibroblasts, and cells of the immune system, including antigen-stimulated T cells and mature dendritic cells. Osteoclast differentiation is initiated via the binding of RANKL to RANK [26]. RANKL-RANK interaction activates the master transcription factor for osteoclastogenesis; nuclear factor of activated T cells, cytoplasmic 1 (NFATc1); tartrate-resistant acid phosphatase; calcitonin receptor; and cathepsin K [27]. OPG is produced by osteoblasts, BMSCs, B cells, and dendritic cells, is a soluble decoy receptor for RANKL, and prevents RANKL from binding to RANK on the surface of osteoclastic lineage cells [28, 29]. The relative ratio of RANKL to OPG is the critical determinant and the final step in the regulation of osteoclast biology and bone resorption. Therefore, dysregulation of the immune system may affect bone remodeling, and the immune system regulates bone metabolism primarily via the RANK/RANKL/OPG signaling pathway [30–33]. Various osteotropic hormones, cytokines, and drugs modulate the RANKL-to-OPG ratio [32, 34, 35].

### 2.2. Immune Cell

#### 2.2.1. T Cells

The primary pathological process of osteoporosis is the imbalance of bone resorption and bone formation during bone remodeling. T cells regulate bone cells and hematopoiesis in the immune system, and these cells secrete inflammatory factors and Wnt ligands to promote bone formation and absorption [2, 3, 36]. T cells also regulate the dynamic balance of metabolism between bone mesenchymal cells and osteoblasts via CD40 ligands and costimulatory molecules [37]. However, the stimulating and inhibiting effects of T cells are related to their subsets, cytokines, and local factors.

T cells may be generally classified as effector-cytotoxic T cells (CD8^+^ cells) and T helper (Th) cells (CD4^+^ cells). CD4^+^ T cells develop into diverse Th cell subsets on activation and expansion and secrete signature cytokine profiles and mediate distinct effector functions [38]. T cells were divided into Th1 or Th2 cells depending on the cytokines produced, with Th1 producing IFN-*γ* and IL-2 and Th2 producing primarily IL-4/IL-5/IL-10, until recently. Regulatory T cells (Tregs, CD4^+^CD25^+^Foxp3^+^) exhibit immune suppressive effects in the immune system, regulate bone remodeling, and are closely linked to skeletal-related disease, such as osteoporosis and rheumatoid arthritis (RA) [39, 40]. Tregs suppress osteoclastogenesis via the production of transforming growth factor- (TGF-) *β*, IL-10, and IL-4 [41, 42] and also regulate osteoclast differentiation via the cytotoxic T lymphocyte antigen (CTLA-4) [43, 44].

A third subset of IL-17-producing effector Th cells, called Th17 cells, was more recently discovered and characterized. Th17 cells play an important role in the pathogenesis of osteoporosis and directly produce high levels of IL-17, RANKL, and TNF to promote the formation and activation of osteoclasts [38, 45, 46]. Th17 cell populations and IL-17 levels were obviously increased in an osteoporosis model of ovariectomized (OVX) animals. Transcription factors related to Th17 cell differentiation are highly expressed [47]. IL-17 significantly promotes the production of RANKL, IL-6, and TNF, which promote bone resorption via upregulation of osteoclast formation [48, 49]. Th17 cells are responsible for regulating osteoclastogenesis, especially in autoimmune arthritis.

#### 2.2.2. B Cells

The role of B cells in osteoimmunological interactions has been a matter of great concern. To our knowledge, B cells are active regulators of the RANK/RANKL/OPG system [23]. Bone-forming osteoblasts and BMSCs have historically been considered an important source of OPG under conditions of physiological bone remodeling. However, some studies demonstrated that B cells and plasma cells in the bone microenvironment are significant sources of OPG [50–52], which is a neutralizing soluble decoy receptor that competes with RANKL, thus blocking the binding of RANKL and RANK, which eliminates the effect of RANKL on osteoclasts [53]. However, other studies also demonstrated that activated B cells contributed to joint destruction via RANKL expression [54–57]. Xu et al. further demonstrated that mechanistic target of rapamycin complex 1 activation-stimulated RANKL expression in B cells sufficiently induced bone loss and osteoporosis [58].

### 2.3. Cytokines

A variety of cytokines are involved in the pathogenic process of osteoporosis. Recent studies showed that women with low bone mineral density (BMD) exhibited a proresorptive cytokine bias [59]. The proresorptive cytokines TNF, IL-6, IL-12, and IL-17 were higher in women with low BMD compared to women with normal BMD, and the antiresorptive cytokines IL-4, IL-10, and IL-23 were lower in peripheral blood mononuclear cells (PBMCs). Estrogen decrease after menopause exerts the same stimulating effect, which increases osteoclastic activity via IL-1, IL-6, and TNF-*α* [60, 61]. IL-1 is a very important cytokine in osteoimmunological processes, and it acts as the primary stimulus of osteoclast-activating factor. TNF-*α* and IL-6 possess the same bone resorbing stimulating activity, and these three cytokines increase the osteoclast response to RANKL, which leads to osteolysis. OPG/RANK/RANKL signaling is the most important vinculum for osteoclastogenesis, and most cytokines exert effects via this pathway. RANKL and macrophage colony-stimulating factor (M-CSF) are the minimal necessary cytokines required for osteoclast formation [61]. M-CSF induces the proliferation and differentiation of osteoclast precursors and improves the survival of mature osteoclasts. Other inflammatory cytokines, such as IL-4, IL-10, IL-12, IL-13, IL-18, and interferon-*γ*, strongly inhibit osteoclastogenesis and reduce bone loss [62–65].

### 2.4. Autoimmune Diseases

Autoimmune diseases, such as RA, ankylosing spondylitis (AS), systemic lupus erythematous, and acquired immune deficiency syndrome, involve the damage to joints and bone. RA is characterized by synovial inflammation that results in severe juxta-articular bone erosions and systemic osteoporosis. Previous research showed a greater risk of osteoporotic fractures in RA patients across all age groups, sex, and various anatomic sites compared to non-RA patients [66]. Lower BMD of the lumbar spine and hip was reported in AS patients compared to a control group [67], and 21% of AS patients exhibited osteoporotic vertebral compression fractures, which was 5 times greater than the control population [68].

Associations between osteoclast and proinflammatory cytokines may partially explain the cause of osteoporosis in autoimmune diseases. Active immune cells at sites of inflammation produce proinflammatory and osteoclastogenic cytokines, which lead to bone erosions and peri-inflammatory and systemic bone loss. Proinflammatory cytokines, such as TNF-*α*, IL-1, IL-6, and IL-17, are higher in RA patients, which increase RANKL expression and cause bone loss [69]. Peri-inflammatory bone formation is impaired, which results in nonhealing of erosions and a local vicious circle of inflammation between synovitis, osteitis, and local bone loss.

## 3. Immunoregulation Mechanisms of Chinese Medicinal Plants in the Prevention and Treatment of Osteoporosis

Many plants in China are used for the prevention and treatment of bone and joint diseases, such as bone pain, osteoporosis, osteoarthritis, and RA. Compounds consisting primarily of these plants, such as Liuwei Dihuang pills (LWDHP) and Zuo-Gui-Wan (ZGW), play important roles in clinical treatment. Many studies investigated the important role in prevention and treatment of osteoporosis [70], and rapid progress has been made in delineating the mechanisms. Therefore, we discuss and summarize the mechanism of some representative Chinese medicinal plants and compounds, especially immunoregulation (Table 1).

### 3.1. Chinese Medical Plants and Its Monomer

#### 3.1.1. *Sinomenium acutum* (*Thunb.*) *Rehd. et Wils.* and SIN

*Sinomenium acutum* (*Thunb.*) *Rehd. et Wils*. has been used as a natural drug to treat rheumatic diseases in China for centuries. SIN was isolated from the root and stem of *Sinomenium acutum* (*Thunb.*) *Rehd. et Wils.* in the 1920s [71], and it exhibited good anti-inflammatory and immunoregulatory properties [72].

Liu et al. [73] found that SIN significantly inhibited [^3^H]-thymidine incorporation into mouse spleen cells activated with concanavalin A. The Th1-specific transcription factor T-bet is selectively expressed in Th1 cells and plays an important role in the development of Th1 cells via initiation of Th1 genetic processes and inhibition of the synthesis of Th2 cytokines. GATA-3 is a Th2 cell-specific transcription factor that is directly involved in the regulation of the differentiation of T lymphocytes, induces the generation of Th2 cells, eosinophil differentiation, and the regulation of eosinophil and natural killer cells; GATA-3 also inhibits the CD8^+^ T lymph cell differentiation and maturation. One clinical trial demonstrated that SIN decreased the expression of T-bet mRNA and IFN-*γ* and regulated the ratio of T-bet and GATA-3 and Th1/Th2 cytokine balance in mesangial proliferative nephriti**s** [74]. The novel derivatives of SIN directly inhibited Th17 cell differentiation and alleviated the inflammatory symptoms of experimental autoimmune encephalomyelitis [75].

SIN is widely used in the treatment of inflammatory diseases, especially RA. SIN significantly improved arthritis in rats via inhibition of synovial fibroblast proliferation and anti-type II collagen antibody levels [76, 77] and regulation of Th1/Th2 and the ratio of MMPs and their endogenous inhibitors, called TIMPs [77, 78]. Recent studies demonstrated that SIN modulated bone metabolism via suppression of osteoclastogenesis and related transduction signals. Li et al. demonstrated that SIN suppressed osteoclast formation and bone loss via modulation of RANKL signaling pathways [79] and Zhou et al. [80] revealed that SIN was a proinflammatory mediator that regulated the immunosuppressive effects of marrow stromal cells and inhibited osteoclast differentiation via inhibition of the PGE_2_-induced OPG/RANKL ratio. Further studies revealed that SIN inhibited the activation and relative gene expression of NF-*κ*B, AP-1, and NFAT and downregulated phosphorylation of MAPK p38 in osteoclastogenesis via reduction of Toll-like receptor 4/TRAF6 expression [81]. Therefore, SIN may be a promising agent for the treatment of osteoporosis.

#### 3.1.2. *Davallia mariesii Moore ex Bak*. and Naringin

Naringin is widely distributed in various types of plants, and it is a major component extracted from the Chinese medicinal herb *Davallia mariesii Moore ex Bak*. This herb exhibits inhibitory effects on inflammatory responses and bone destruction and anabolic effects on bone in clinical treatment. The experimental results confirmed that naringin inhibited inflammation via reduction of inflammatory cytokine and NF-*κ*B expression [82].

Naringin exhibited some therapeutic effects on osteoporosis in animal experiments. Naringin inhibited tartrate-resistant acid phosphatase activity, decreased RANKL expression, inhibited osteoclast activity, reduced bone loss, and decreased BMD, which altered bone resorption. Further study demonstrated that naringin inhibited osteoclast activity via inhibition of RANK-mediated NF-kappaB and extracellular regulated protein kinase signaling [83]. Naringin increased osteoblast proliferation by increasing BMP-2 expression via PI3K, Akt, c-Fos/c-Jun, and the AP-1 pathway [84]. Wong et al. and Pang et al. also found that naringin increased osteopontin and osteoprotegerin expression and osteocalcin [85, 86]. Naringin promoted the osteogenic differentiation of BMSCs by upregulating Foxc2 expression via the IHH signaling pathway [87].

#### 3.1.3. *Epimedium davidii Franch.*, Icariin, and Icaritin

*Epimedium davidii Franch.* has been used for centuries to treat osteoporosis, based on the function of “strengthening” bones. Icariin is the primary active flavonoid glucoside isolated from *Epimedium davidii Franch.*, and it is highly related to the therapeutic effects of this herb. However, its bone-strengthening activity attracted much more attention in recent years. Icariin enhances antiosteoporotic activity by initiating osteoblastic differentiation and mineralization, inhibiting adipogenesis, preventing osteoclast differentiation, and inducing apoptosis of osteoclasts, which decrease bone resorption and bone loss [88–92]. Liu et al.'s research indicated that icariin suppressed the differentiation of mesenchymal stem cells into adipocytes via inhibition of peroxisome proliferator-activated receptor gamma, CCAAT/enhancer binding protein *α*, fatty acid-binding protein 4 mRNA, N1ICD, and jagged1 protein expression and increasing Notch2 mRNA in OVX rats, which improved osteoporosis [93]. Further research indicated that icariin also promoted bone fracture healing in OVX osteoporotic rats [94, 95]. Icariin could increase the proliferation and matrix mineralization of osteoblasts and promote NO synthesis. Further study showed that with icariin treatment, the BMP-2, SMAD4, Cbfa1/Runx2, and OPG gene expressions were upregulated; the RANKL gene expression was however downregulated. This effect may contribute to its action on the induction of osteoblast proliferation and differentiation, resulting in bone formation [96]. Liang's results also indicated that icariin could promote bone formation via the BMP-2/Smad4 signal pathway in hFOB 1.19 cells then promote bone formation [97].

Icaritin, an internal metabolite of icariin, possesses similar function with icariin. Icaritin inhibited osteoclast formation via the downregulation of TRAF6 and coordinated inhibition of NF-*κ*B, MAPK/AP-1, and reactive oxygen species signaling pathways to reduce NFATc1 expression and activity in vitro and in an OVX rat [98]. Wu et al. and Sheng et al. indicated that icaritin enhanced the osteogenic differentiation of derived mesenchymal stem cells and human adipose tissue-derived stem cells by increasing the protein levels of BMPs, Runx2, and osteocalcin [99] and inhibiting adipogenesis of marrow mesenchymal stem cells via suppression of glycogen synthase kinase-3*β* and peroxisome proliferator-activated receptor gamma [100].

#### 3.1.4. *Eucommia ulmoides* and Quercetin

*Eucommia ulmoides* is a kidney-tonifying herbal medicine in China with a long history for treatment of bone and joint diseases, such as osteoporosis, osteoarthritis, and RA. *Eucommia ulmoides* and its extracts participate in bone metabolism via activation of osteoblasts to facilitate osteogenesis and suppression of osteoclast activity to inhibit osteolysis [101]. In vivo studies showed that extracts of *Eucommia ulmoides* improve bone biomechanical quality via modification of BMD and trabecular microarchitecture in OVX rats [102–105]. *In vitro* evidence suggests that extracts of *Eucommia ulmoides* could induce primary osteoblastic cell proliferation and differentiation and inhibit osteoclastogenesis via an increase in OPG and decrease in RANKL expression [106].

An ethanol extract of *Eucommia ulmoides* relieved arthritis degradation via inhibition of the key proinflammatory cytokines TNF-*α*, IL17, and IL-1*β* and increases the anti-inflammatory effects of IL-10 [107]. The PI3K/Akt pathway may play an important role in this process. *Eucommia ulmoides* also inhibited the progression of osteoarthritis via inhibition of the PI3K/Akt pathway to reduce inflammatory cytokines, bone destruction, and bone loss [108, 109].

The flavonol glycoside quercetin was isolated from the leaves of *Eucommia ulmoides*, and its structure was identified using NMR and ESIMS analyses [110]. Quercetin exhibited soluble epoxide hydrolase inhibitory activity and anti-inflammatory properties, partially because of its capacity to downmodulate the NF-*κ*B signal transduction pathway [111–114]. Quercetin suppressed osteoclastogenesis in vitro and prevented bone loss in OVX mice in vivo [115, 116]. Yamaguchi and Weitzmann found that quercetin potently suppressed osteoclastogenesis and RANKL-induced NF-*κ*B activation in osteoclast precursors [117]_._ Guo et al. also found that quercetin inhibited RANK, TRAF6, and COX-2 expression, induced apoptosis, and inhibited bone resorptive activity in LPS-induced mature osteoclasts. Quercetin promoted the apoptotic signaling pathway, including increasing the phosphorylation of p38-MAPK, c-Jun N-terminal kinases/stress-activated protein kinases (JNK/SAPK), and Bax and inhibited Bcl-2 expression [118]. Some studies demonstrated that quercetin exerted a stimulatory effect on bone formation and reversed the inhibition of osteoblast differentiation induced by LPS via MAPK signaling in vitro [119, 120].

#### 3.1.5. Other Chinese Medicinal Plants and Monomers

Many Chinese medicinal plants and monomers effectively treat osteoporosis. Echinacoside is isolated from *Cistanchetubulosa* (*Schrenk*) *R. Wight* stems, and it exhibited notable antiosteoporotic effects in an OVX rat model of osteoporosis and enhanced bone regeneration in MC3T3-E1 cells in vitro [121]. *Radix Dipsaci* has long been used as an antiosteoporotic drug. Radix Dipsaci total saponins are the main active ingredient of *Radix Dipsaci*, and this component effectively suppressed the loss of bone mass in OVX rats [122]. *Achyranthes bidentata Blume.* improved bone biomechanical quality via modifications of BMD6 and trabecular microarchitecture, which prevented OVX-induced osteoporosis in rats [123]. *Achyranthes bidentata Blume.* and Panax notoginseng saponin are the main active components of *Panax notoginseng* that prevent OVX-induced osteoporosis via enhancement of BMD, bone strength, and prevention of the deterioration of trabecular microarchitecture in OVX osteoporosis rats [124]. Diarylheptanoid from *Curcuma comosa Roxb.* decreased NFATc1 and c-Fos expression via the MAPK pathway and inhibited RANKL-induced osteoclast differentiation [125]. The natural compound *Polygonatum sibiricum* polysaccharide promoted osteoblastic differentiation and mineralization via the ERK/GSK-3*β*/*β*-catenin signaling pathways [126]. These studies provide a therapeutic approach for the use of Chinese plants in the prevention of osteoporosis.

### 3.2. Compound Recipes of Medicinal Plants

#### 3.2.1. LWDHP

As a classic formula of traditional Chinese medicine, LWDHP has been used to prevent and treat various diseases with characteristic features of kidney yin deficiency in China for more than 1000 years. LWDHP is widely prescribed as therapy or adjuvant therapy for various diseases, such as menopause, osteoporosis, and diabetes. LWDHP exhibits a wide range of pharmacological effects via regulation of the balance of the neuroendocrine immunomodulation network [127].

LWDHP exhibits bidirectional regulation to the immune system. LWDHP exerted its pharmacological action via immunomodulation rather than immunosuppression, which differs from Cy and CsA [128]. Jian et al. demonstrated that LWDHP significantly inhibited the mRNA expression of IL-1 (Th1 cytokines) and promoted the expression of IFN-*γ* (Th1 cytokines), IL-4 (Th2 cytokines), and IL-10 (Th2 cytokines) in splenocytes of AA rats. These results indicated that LWDHP enhanced the function of splenic Th cells via restoration of Th1 and Th2 cytokine levels and modulation of the balance of the Th1/Th2 [129]. LWDHP also significantly improved the function of T and B lymphocytes in aging mice and corrected the imbalance of CD4^+^/CD8^+^ T cells in the spleen.

The mechanism of LWDHP treatment of osteoporosis is also related to its immunomodulatory function. The JAK/STAT signaling pathway plays a vital role in bone metabolism. LWDHP regulates immune function by increasing the expression of the immune-related gene cardiotrophin-like cytokine factor 1 and activation of the JAK/STAT signaling pathway to treat postmenopausal osteoporosis with kidney yin deficiency [130]. Xia et al. suggested that LWDHP could alleviate osteoporosis partially via a significant enhancement in the levels of Lrp-5, *β*-catenin, Runx2, and Osx, which were involved in the canonical Wnt/*β*-catenin signaling pathway of osteoblasts, in osteoporosis model induced by ovariectomy and in vitro experiments [131].

#### 3.2.2. ZGW

The ZGW is another classical traditional Chinese medicine (TCM) herbal prescription for tonifying kidney yin. This prescription was recorded in Jing yue Quan shu, which is a famous TCM book, in 1624 A.D.

Our results showed that the ZGW significantly increased the expression levels of Wnt1, low-density lipoprotein receptor-related protein 5, and beta-catenin proteins in osteoblasts and BMSCs of glucocorticoid-induced osteoporosis rats after eight weeks of administration [132]. These results indicated that the ZGW prevented and treated osteoporosis, and the Wnt signal transduction pathway played an important role in this progress. The Th17/Treg paradigm plays a vital role in the regulation of bone metabolism. Lai et al. found that the ratio of Th17 and Treg shifted to Th17 in estrogen-deficient OVX and aged mice. Treatment with ZGW markedly enhanced BMD, decreased IL-6 and ROR*γ*t expression, and significantly increased the level of Foxp3. These results indicated that the ZGW could prevent bone loss, and the mechanism was a Th17/Treg paradigm that skewed towards Treg [133].

## 4. Conclusion

Many studies investigate the pathogenesis of osteoporosis, and the immunological regulation of osteoporosis is a new research hotspot that provides a new mechanism of the pathogenesis of osteoporosis. Immune and bone cells exist together in the microenvironment of bone cavities, and these cells share a variety of regulatory molecules. Many cytokines in bone metabolism play a very important role in the proliferation, differentiation, and activation of osteoclasts and osteoblasts. However, the specific mechanisms of these cytokines have not reached an indisputable conclusion, and the role of many cytokines in the process of bone metabolism is not clear.

The clinical efficacy of Chinese medicinal plants has shown their advantages in the treatment of osteoporosis. An increasing number of studies demonstrated that these plants play an important role in the treatment of osteoporosis by regulating the immune system, but more research is required. Notably, the mechanism of action of traditional Chinese medicine is not a single pathway, but it is multiroute and multitargeted. Therefore, its regulation of the immune system cannot be the only mechanism for the treatment of osteoporosis. Our continued improvement in understanding of the immunoregulatory mechanisms of osteoporosis will further elucidate the immunoregulatory mechanism of Chinese medicine for the treatment of osteoporosis.

## Figures and Tables

**Table 1:** Chinese medicinal plant targets involved in immunoregulation effects.

Chinese medicinal plants or compounds	Pharmacological effects	Target factors	Action	Model	Reference
SIN	Immunoregulation	T-bet/GATA-3	T-bet/GATA-3 ratio ↓ from PBMCs, IFN-gamma in the serum ↓	MsPGN	[74]
1032 (a derivative of SIN)	Inflammatory reaction	IL-17	IL-17, IL-6, TNF-alpha ↓, I*κ*B*α* ↑, suppression of Th17	Encephalomyelitis/dendritic cells	[75]
SIN	Immunoregulation	MMPs/TIMPs	Synovial fibroblast proliferation, anti-type II collagen antibodies, IL-1*β*, IL-6, IL-5, TGF-*β* ↓, the ratio of MMPs/TIMPs ↓	Arthritis rats	[76–78]
SIN	Inflammatory reaction	TLR4/TRAF6	TNF-*α*, TLR4, TRAF6, Fra-1, MMP-9, NF-*κ*B, AP-1, NFAT, MAPK p38 ↓	Lipopolysaccharide-induced osteoclastogenesis and osteolysis	[81]
Naringin	Inflammatory reaction	Inflammatory cells	Inflammatory cells, high-mobility group box-1 ↓	Arthritis mice	[82]
Icaritin	Immunoregulation	TRAF6	NFATc1, TRAF6 ↓	OVX rat/RAW 264.7 mouse monocyte cell line/human PBMC	[98]
*Eucommia ulmoides*	Inflammatory reaction	Inflammatory cytokines	TNF-*α*, IL17, IL-1*β* ↓, IL-10 ↑	Arthritis rats	[107]
*Eucommia ulmoides*	Inflammatory reaction	PI3K/Akt, inflammatory cytokines	IL-1*β*, IL-6, MMP-3, phosphorylated MAPKs, PI3K/Akt, GSK-3*β*, NF-*κ*B ↓, Nrf2, HO-1 ↑	Osteoarthritis rats/LPS-stimulated BV-2 microglial cells	[108, 109]
*Quercetin*	Inflammatory reaction	NF-*κ*B	IL-6, IL-1*α* ↓, IL-3, IL-4 ↑	RAW 264.7 cells	[112]
LWDHP	Immunoregulation	Inflammatory cytokines	IL-2 ↓, interferon-gamma, IL-4, IL-10 ↑	Adjuvant arthritis rats	[129]
ZGW	Immunoregulation	Th17/Treg	IL-6, ROR*γ*t ↓, Foxp3 ↑	Estrogen-deficient mice	[133]

Sinomenine (SIN); mesangial proliferative glomerulonephritis (MsPGN); matrix metalloproteinases (MMPs); tissue inhibitors of metalloproteinases (TIMPs); tumor necrosis factor receptor associated-factor 6 (TRAF6); Fos-related antigen-1 (Fra-1); activator protein-1 (AP-1); nuclear factor of activated T cells (NFAT) c1; mitogen-activated protein kinases (MAPK); lipopolysaccharide (LPS); Toll-like receptor 4 (TLR4); phosphoinositide-3 kinase (PI3K); nuclear factor-*κ*B (NF-*κ*B); glycogen synthase kinase-3*β* (GSK-3*β*); nuclear factor erythroid 2-related factor 2 (Nrf2); heme oxygenase-1 (HO-1), related orphan receptor gamma t (ROR*γ*t).
